# The Physicians of Oldham County, Ky.

**Published:** 1875-11

**Authors:** 


The Physicians of Oldham County, Kentucky, issued a circular setting forth the grievances of the profession in the way of long credits, and a very large percentage of unpaid and lost bills as the natural consequence, and calling for reform in this particular.

Upon the basis of this circular, the editor of the *Cincinnati Lancet and Observer* thus sensibly lucubrates and scintilates:

"It means pelf—yea, more pelf; it has pelf running all through it from the beginning to the end, and to all of which we say amen, for verily pelf is a good thing to have. It is useful both in the house and out of the house; it makes a man's clothes fit better, keeps his wife, bairns and horse shod; it greases all the cogs, reduces friction to a minimum amount, smoothes out the crows' feet, and keeps off wrinkles. As a hypnotic, it is generally more efficacious than opium; as a nervous sedative, it is twice as good as bromide of potassium or asafœtida; as a tonic, it can't be beat; as an aphrodisiac, it will strengthen up the most worn-out old *roue* in less than half the time it would take under full doses of damiana or phosphorus. It is the most soothing panacea that was ever applied to a weak back. Piles—of pelf are good; they assist digestion; in fact, are an indication of good health.

"For the instantaneous relief of intense pain caused by tick, we can conscientiously recommend pelf; it is altogether superior to deep injections of chloroform, and any sloughing that may be produced is of a laudable character. The virtues of pelf, when made into a plaster that will stick, are past all comprehension. Get some and try it.

"The idea that a young man is to spend years of time, and probably all of his money, in acquiring a highly honorable and dignified profession, and then to have his dignity braced up with an empty stomach or one filled with husks, is all bosh. Pelf is the only remedy. If you have earned some, make your demand for it at once. Your patrons are just as much in duty bound to pay you as soon as your services are rendered as they are to pay the grocer, carpenter, blacksmith, or farm hand, and no more; neither has a right to be recognized as a preferred creditor over the other. Credit means unpleasantness, loss, and probably ruin. Cash means adipose, a happy wife, and rolicking babies.

"There is nothing unprofessional in the act of suing a man for your dues, if he is able to pay and refuses to come down with his pelf in any other way. Nor is it a violation of the Code of Ethics to accept a retaining fee, either large or small. * * * Gentlemen of the medical profession in Oldham and adjoining counties in Kentucky, you are on the right track. Go ahead, and just so surely will the Lord take a liking to you. Solemnity is good, seriousness is good, and dignity is good; but when these graces are honestly earning and getting pelf, their value is inconceivably enhanced."

Why do not physicians promptly collect their dues? All admit the justice, the propriety, nay, the necessity of such a course, as a matter of good business policy. Why is it not done?

It is a well accepted observation that medical men, as a class, are notoriously loose jointed as business men. Some of them are constitutionally so; they are indolent, careless, wanting in system and in business tact; they are content to live along from year to year with scanty support, broken down in credit, eking out a hand to mouth subsistence. Such men are to be found in every avenue of life, in all occupations and callings; they are by no means peculiar to the medical profession. We must look elsewhere for a solution of the problem we have propounded. Let us examine a little this movement for prompt pay in Kentucky:

Upon the first of August last the physicians of Oldham county issued a circular addressed to their brethren in the surrounding region, setting forth in strong terms their grievances, their "*financial distress, poverty*, and all their attendant evils. We feel that this *glaring injustice* to ourselves has gone too far *unchecked!*"

"Therefore we *most earnestly* solicit that you, with every other professional brother in striking distance, meet with us in *solemn and serious convention*, at LaGrange, on Tuesday, the 17th day of August, for the purpose of consulting together on our financial difficulties, and of uniting upon some SURE METHOD of collecting our bills at *regular and short intervals*, and of thus placing ourselves upon an equal footing with the other trades and callings of our country." (!!) "We therefore call upon every doctor to come up to this convention, and show his *honor and manhood* in sustaining the *independence and dignity* of his profession."

Bravo! said we. Bravo! bravo!! resounded from every hamlet and cross-roads throughout the South. Truly did the mountain labor, and as truly do we behold its issue—the little mouse:

"RESOLVED, By the physicians of Oldham and adjoining counties, that we will, in future, be more diligent in the collection of our fees, and we *believe we should inform our patrons*, by letter or otherwise, at least *once in six months*, the *amount of our claims* against them.(!)

"RESOLVED, That all medical fees are due when the services are rendered, and that each physician should present his bill for collection *as soon as he thinks his interests* may require.(!!)

"RESOLVED, That the custom of giving medical services to ministers and their families free of charge be dispensed with, and that hereafter they be charged as other persons, *according to circumstances*."

Is not the trouble in Oldham the same as elsewhere? Is it not *the profession themselves who are starving themselves*, instead of an ungrateful community for whom they labor? Are these gentlemen in earnest? Do they mean to stand up to each other in manly defense of their rights? *Dare they trust each other to take a single step forwards for their common protection and emolument?*

Well do we remember our participation, years ago, in a local medical convention for similar purposes to that of Oldham, which passed the usual *resolutions*, and in terms more emphatic and far less equivocal than the latter. Well do we remember our earnest efforts to check the zeal, laudable as it appeared to be, of two members who had been notorious for low charges, but who had now swung to the opposite extreme, and, in the adjustment of a fee bill, labored to put up the items to double the sums which had been customary. When a good average rate of charges was settled upon and published, blasted were our new born hopes, and sad our disappointment, to find these zealous disciples, in spite of their pledges, returning, like a dog to his vomit, and again dividing the established fees by two. When the great medical fraternity will learn lessons of the higher orders of brute creation and *refuse to devour the flesh of their own species*, then may we hope to rid ourselves of the impecuniosity which so sadly afflicts us.

